# Infantile Fibrosarcoma in a Child: a Case Report

**Published:** 2013-07-22

**Authors:** A Hashemi, S Tefagh, A Seifadini, M Moghimi

**Affiliations:** 1 Department of Pediatrics, Hematology, Oncology and Genetic Research Center, Shahid Sadoughi University of Medical Sciences Health Services, Yazd, Iran.; 2 MD, Hematology, Oncology and Genetics Research Center, Shahid Sadoughi University of Medical Sciences and Health Services, Yazd, Iran.; 3 Medical Student, Islamic Azad University branch Yazd, Yazd, Iran.; 4 Department of Pathology, Shahid Sadoughi University of Medical Sciences Health Services, Yazd, Iran.

**Keywords:** Infant, Fibrosarcoma, Report

## Abstract

**Background:**

Infantile Fibrosarcoma is a rare soft tissue tumor in infants and children mostly located in extremities. An infantile and adult form has similar histopathological patterns but survival prognosis is much better in infantile form. Recurrence of infantile fibrosarcoma is common but the rates of metastasis are less than 10 percent in children younger than five Years and 50 percent in children more than 10 years old.

**Case presentation:**

In this case report, we presented a nine years girl with a relapsing mass in her left hand. The pathologic findings showed sheets of spindle-shaped cells with suggested diagnose of infantile fibrosarcoma. She was successfully treated with combination of surgery and chemotherapy with a good outcome.

**Conclusion:**

Infantile fibrosarcoma is a differential diagnose of soft tissue mass in infants and children. It has a good prognosis and distant metastasis is uncommon. Choice of treatment is surgery but chemotherapy and radiotherapy were useful in decrease metastasis.

## Introduction

Infantile fibrosarcoma is a rare early childhood malignancy. It includes aproximately 10 % of all sarcomas in children (1). It is mostly presented as a tumor in extremities, trunk, head and neck. Ultimate diagnosis is made by physical examination, special radiologic studies, and biopsy (2). Infantile fibrosarcoma is spindle cell tumor originated from soft tissue. Histologically, it is similar to adult-type but with a better prognosis. In the Pediatric Oncology Group classification of NRSTS, infantile fibrosarcoma is classified as a grade I lesion. Although recurrence of infantile fibrosarcoma is common, metastatic spread is infrequent(1). Surgery is considered the main choice of treatment. The use of adjuvant therapy and chemotherapy is not clear yet, but in most of high grade tumors are used for treatment of microscopic metastasis (3). In comparison with adults, chemotherapy is more effective in children(4). This case report, presented a nine years old girl with a mass in her hand which was relapsed locally. The tumor location, radiological and pathological findings proved the diagnosis of fibrosarcoma. In this age, infantile fibrosarcoma is rare and generally misdiagnosed because of histological similarities to benign tumors.

## Case report:

A nine years old girl was presented with a mass in her left hand in polmar side. Initially, her mass was noticed four years ago with 3*4cm size which was growing gradually. After excision surgery, it was diagnosed as soft tissue mass (lypoma). She was referred to Shahid Sadoughi clinic of pediatric oncology due to the relapse of the tumor four years later. On physical examination, a firm, immobile mass with mild tenderness and discrete borders was palpable. Her left finger movement was limited in all ranges. No other abnormalities were found in the examinations. Laboratory tests were normal.

Magnetic Resonance Imaging (MRI) findings showed 4*5cm neoplast lesion in left hand. Deformity of left hand was presented. Fifth metacarpal bone was not seen. Invasive soft tissue mass (recurrence or remained sarcoma) was suggested in MRI results.

**Figure1:**
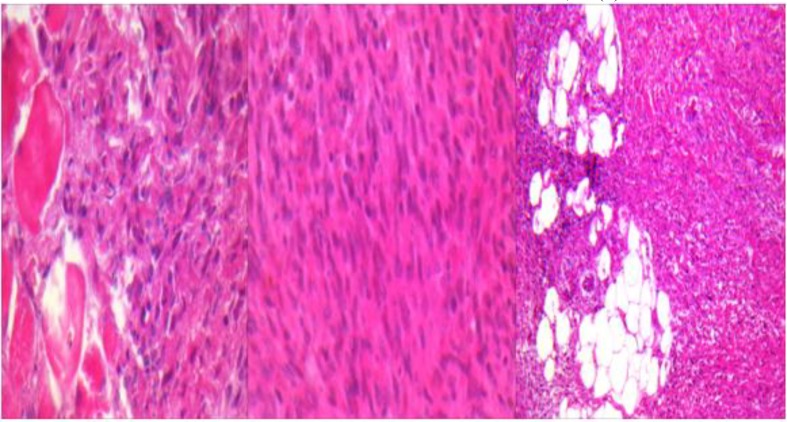
Infantile Fibrosarcoma with High mitotic figures

The histology of the mass was reanalyzed because of the relapse. The findings showed sheets of spindle-shaped cells with suggested diagnose of infantile fibrosarcoma. Afterwards, excisional surgery was do and pathological report revealed cellular spindle neoplastic tissue with fasicular pattern of slightly pleomorphic tumoral cells with vesicular nuclei and occasionally prominent nucleoli admixed by foci of hemangiopericytoma like pattern. Tumoral cells infiltrated into fatty tissue, bone and skeletal muscle. In some focus more than five mitotic figures in per HPF were also seen moderate infiltrations of chronic inflammatory cell with follicular aggregation. (Figure, 1). Base on these findings infantile fibrosarcoma was the diagnosis. Metastatic work up was negative. Abdomen and pelvis scan with IV (intravenous) and oral contrast and thorax scan with IV contrast were normal. Abdomen and chest ray were normal.

After the surgery, the treatment was completed with systemic chemotherapy using oncovin, actinomycin and endoxan.

High response to chemotherapy was observed. No relapse was observed up to now.

## Discussion

The main cause of infantile fibrosarcoma still remains unknown, but some gene fusions due to translocation and some trisomies have been reported(5). Infantile fibroracoma includes less than one percent of childhood tumors and about 10 percent of soft tissues sarcomas(1,6).

Infantile fibroracoma usually presents in the first five years of life, most of them under three years and about 40 percent under three months(7,8). It is rarely appears at older ages between 10 to 15 years old (9,10). In this case the patient was nine years old when diagnosed and the tumor was found 4 years earlier.

Clinically the most common sign is a local, progressive mass with no discrete borders in distal part of extremities. In some cases, the surface is necrotic or ulcertic which makes the appearance similar to vascular malformations like hemangioma(4). Also it may be misdiagnosed as other soft tissue tumors as it was misdiagnosed with lypoma in this case. MRI is a good choice for better evaluation in diagnoses and follow-up of soft tissue masses. MRI is considered the modality of choice for evaluation of the diseases in the extremities, head, neck and pelvis(11,12). In this case, MRI results were suggestive of soft tissue sarcoma. Although most of the tumors have a rapid growth manner, in this case, the growth of tumor was slow.

Two types of infantile fibroracomas are: Desmoplastic type and medullary type. Desmoplastic type in children resemble to adults (1). The histological diagnosis of fibrosarcoma may sometimes be difficult.

 Local recurrence is reported in 7-34 percents of patients. The chance of recurrence is much more in older than younger ones. In this case, recurrence occurs after 4 years.

Distant metastasis is uncommon and was reported only in four percent of cases at the beginning of diagnoses. Children who are less than 5 years old are at the risk of relapse of tumor, whereas the incidence of metastases is under 10% in these patients. The rate of metastatic spread in children who are 10 years old or older is 50% at 5-year follow-up(13). In our patient no metastasis was found.

Infantile fibroracoma has a good prognosis. A five-year survival is reported 80 to 100 percent(14).

Currently, the main treatment in most cases is surgery with wide local excision which sometimes leads to amputation. Radiotherapy and chemotherapy were shown to be useful in reduction of tumors bulk especially in metastatic, relapsing and un-respectable tumors. In older children chemotherapy is offered to decrease the possibility of metastasis (11). In this case, surgery was followed by chemotherapy for two reasons: 1.the child was nine years old 2.the tumor was replacing. After treatment, complete remission was obtained.

The effective regimen for chemotherapy is vincristine, actinomycin D and cyclophsphamide. Orbach et al reported that 71% of patients with infantile fibrosarcoma responded to vincristin-actinomycin-D chemotherapy. The 5-year survival in these patients was 89% (15).

## Conclusion

Although infantile fibrosarcoma is rare and usually presented after birth, but it is necessary to consider it as a differential diagnose of soft tissue mass in infants and even in children. Patients should be followed up for detecting further relapse or metastasis especially in older ages.
